# Effect of *Uncaria tomentosa* Extract on Apoptosis Triggered by Oxaliplatin Exposure on HT29 Cells

**DOI:** 10.1155/2014/274786

**Published:** 2014-11-11

**Authors:** Liliane Z. de Oliveira, Iria Luiza G. Farias, Melânia L. Rigo, Werner G. Glanzner, Paulo Bayard D. Gonçalves, Francine C. Cadoná, Ivana B. Cruz, Júlia G. Farias, Marta M. M. F. Duarte, Luzia Franco, Gustavo Bertol, Elisangela Colpo, Patricia C. Brites, João Batista T. Rocha, Daniela B. R. Leal

**Affiliations:** ^1^University Hospital of Santa Maria, Federal University of Santa Maria (UFSM), Avenida Roraima, Prédio 20, 97105-900 Santa Maria, RS, Brazil; ^2^Postgraduate Program in Pharmaceutical Sciences, UFSM, 97105-900 Santa Maria, RS, Brazil; ^3^Laboratory of Biotechnology and Animal Reproduction, UFSM, 97105-900 Santa Maria, RS, Brazil; ^4^Postgraduate Program in Biochemistry and Toxicology, UFSM, 97105-900 Santa Maria, RS, Brazil; ^5^Postgraduate Program in Agronomy, UFSM, 97105-900 Santa Maria, RS, Brazil; ^6^Lutheran University of Brazil (ULBRA), BR 287, Km 252, 97020-001 Santa Maria, RS, Brazil; ^7^Herbarium Laboratório Botânico Ltda., Avenida Santos Dumont 1100, 83403-500 Colombo, PR, Brazil

## Abstract

*Background/Aim*. The use of herbal products as a supplement to minimize the effects of chemotherapy for cancer treatment requires further attention with respect to the activity and toxicity of chemotherapy. *Uncaria tomentosa* extract, which contains oxindole alkaloids, is one of these herbal products. The objective of this study was to evaluate whether *Uncaria tomentosa* extract modulates apoptosis induced by chemotherapy exposure. *Materials and Methods*. Colorectal adenocarcinoma cells (HT29 cells) were grown in the presence of oxaliplatin and/or *Uncaria tomentosa* extract. *Results*. The hydroalcoholic extract of *Uncaria tomentosa* enhanced chemotherapy-induced apoptosis, with an increase in the percentage of Annexin positive cells, an increase in caspase activities, and an increase of DNA fragments in culture of the neoplastic cells. Moreover, antioxidant activity may be related to apoptosis. *Conclusion*. *Uncaria tomentosa* extract has a role for cancer patients as a complementary therapy. Further studies evaluating these beneficial effects with other chemotherapy drugs are recommended.

## 1. Introduction

The association among reactive oxygen species (ROS), cellular oxidative stress, and cancer risk has been well established, resulting in studies demonstrating that the use of herbal products with antioxidant properties can reverse or minimize these negative effects, thereby improving the health of individuals. However, the nature of this association is complex and at times may appear paradoxical [1]. In 2006, Schumacker [2] summarized this relationship in five points: (1) oxidative stress and ROS can cause cancer, (2) transformed cells generate more ROS compared with normal cells, (3) antioxidant systems, such as thioredoxin and superoxide dismutase (SOD), are enhanced in certain malignant cells, (4) stimulation of cell cycle progression by growth factors or mutations, which activate signaling via the tyrosine kinase receptor, involves an increase in ROS, and (5) various chemotherapeutic agents may be selectively toxic to tumor cells because they increase oxidative stress and drive already “stressed” cells beyond their limit. Highlighting the relationship to oxidative stress, H_2_O_2_ levels in particular play a key role in the induction of apoptosis. In this way, antioxidant enzymes such as Mn-SOD, which directly affect the levels of these elements, are crucial in both apoptosis and tumor cell resistance to chemotherapy. An increase in Mn-SOD in colorectal carcinomas contributes to the resistance and insensitivity of tumor cells in response to therapy, resulting in a worse prognosis [3]. Moreover, Hussain and coworkers [4] demonstrated that apoptosis induced by the p53 gene in lymphoblasts exposed to chemotherapy was mediated by an increased expression of Mn-SOD. Priego and coworkers [5] confirmed a key role of Mn-SOD in the elimination of colon carcinoma cells (HT29) in animals treated with a FOLFOX regimen (oxaliplatin and 5-fluorouracil).

*Uncaria tomentosa* (Willd) DC., Rubiaceae, which is also known as cat's claw, contains more than 50 chemical constituents, including oxindole and indole alkaloids, polyphenols (flavonoids, proanthocyanidins, and tannins), glycosides, triterpenes derivatives quinovic and quinic acid, and saponins [6]. Among these compounds, oxindole alkaloids (pentacyclic (POA) and tetracyclic (TOA)) have been recognized as phytochemical markers of this species due to several pharmacological activities [6–8]. However, the chemical composition of this agent may vary according to geographical region and seasons [6]. Thus, some plants have higher contents of POA (speciophylline, uncarine F, mitraphylline, isomitraphylline, pteropodine, and isopteropodine), while others have higher contents of TOA (rhynchophylline and isorhynchophylline). Among the pharmacological properties of* Uncaria tomentosa* is antioxidant activity, with an increased activity of superoxide dismutase enzyme (SOD) [9]. Aqueous/hydroalcoholic extracts have demonstrated myeloproliferative effects [10, 11], induced apoptosis in neoplastic cells [12–14], as well as its POA isolates [15], and exhibited antitumor effects [16]. In a previous study using* Allium cepa *cytogenetic analysis, we demonstrated that dry* Uncaria tomentosa* hydroalcoholic extract exerted no mutagenic effects and thus exhibited antimutagenic potential, reducing the DNA damage induced by chemotherapy exposure, particularly anaphase-telophase chromosome aberrations. A reduction in oxaliplatin-induced lipid peroxidation was also observed.

Given that apoptosis caused by chemotherapy is mediated by free radicals, we examined whether* Uncaria tomentosa* extract could affect this activity, due to its antioxidant properties. Similarly, we analyzed whether the extract promoted chemotherapy-induced DNA repair in tumor cells, as previously observed in cytogenetic studies using* Allium cepa*.

## 2. Materials and Methods

### 2.1. Cancer Cell Lines

HT29 cells were obtained from Banco de Células do Rio de Janeiro—BCRJ (Code BCRJ: 0111). HT-29 cells were grown in DMEM-F12 (Sigma Aldrich; pH 7.4) supplemented with 10% FCS (Gibco/Invitrogen). Cultures were maintained at 37°C in a humidified atmosphere with 5% CO_2_. The cells were harvested by incubation for 5 min with 0.05% (w/v) trypsin (Sigma) in Hanks buffered solution (Gibco/Invitrogen) containing 0.3 mmol/L EDTA followed by the addition of 10% FCS to inactivate the trypsin. Cells were quantified using a Neubauer chamber, and cellular viability was assessed using the trypan blue exclusion method.

### 2.2. Cell Treatments

Cells were seeded in 6- or 96-well flat bottom plates (TPP) at 1 × 10^6^ or 0.5 × 10^5^ viable cells/mL, respectively, and were allowed to adhere overnight at 37°C. The cells were treated with the following concentrations of cytotoxic agent for 24, 48, or 72 h:

1. control: culture medium without* Uncaria tomentosa *extract or oxaliplatin,
2. chemotherapy: oxaliplatin at 20 *μ*mol/L,
3. *Uncaria tomentosa* extract at 750 *μ*g/mL,
4. *Uncaria tomentosa* + chem.:* Uncaria tomentos*a extract at 750 *μ*g/mL + oxaliplatin at 20 *μ*mol/L.

### 2.3. Reagents

Oxaliplatin (DACH-(oxalate) platinum II, L-OHP) (Oxaliplatina) was purchased from Eurofarma.* Uncaria tomentosa* extract (5%) was prepared by ultra-turrax extraction (Biotron-Kinematica AG) of ground bark of plants from Peru (Naturex) with 70% ethanol (Dipalcool) and identified according to the methodology of USP 36. The fluid was centrifuged (Centrifuge Suzuki), concentrated in a heating tank (MCA-ALW) to remove the alcohol, and spray-dried (Kohls) using silicon dioxide (Evonik) and microcrystalline cellulose 102 (Blanver) as excipients.

### 2.4. Analysis of Dry* Uncaria tomentosa* Extract

The following reagents were used: acetonitrile (JTBaker), triethylamine (Fluka), acetic acid (JTBaker), polyamide (Fluka), ethanol (Vetec), and ultrapure water. Sample extraction was performed using a Unique ultrasound, model USC 5000A, at 40 kHz. Chromatographic analyses were performed on the Agilent 1100 HPLC system and a Zorbax XDB C-18 column (150 mm × 4.6 mm, 3.5 *μ*m Agilent) at 15°C. Samples (80 mg) were diluted in 60% ethanol (10 mL) and subjected to sonication (20 min at 30°C). Next, 2 mL of sample was passed through a column containing 200 mg of polyamide, and the eluate was injected into an HPLC system. Separation was achieved using gradient elution of water (0.2% acetic acid) adjusted to pH 6.9 with triethylamine (A) and acetonitrile (B) at a flow rate of 0.8 mL/min, detection was performed at 245 nm, and the concentration of oxindole alkaloids (OA) was calculated as previously described in Bertol and coworkers [17].

### 2.5. Flow Cytometry Analysis

PE Annexin V versus 7-amino-actinomycin D (7-AAD) staining was performed according to the manufacturer's instructions (BD Biosciences) using PE Annexin V Apoptosis Detection Kit I and analyzed by flow cytometry. A total of 10,000 events were acquired and analyzed using FACSCalibur with CELLQuest software. Cells live-gated cells within the Annexin-V^+^7AAD^−^ compartment were identified as early apoptotic cells and gated cells within the Annexin-V^+^7AAD^+^ compartment were identified as late apoptotic/dead cells.

### 2.6. Cells Mortality Assay

DNA fragments present in the cell culture medium indicated cell death. We used the Quant-iT PicoGreen dsDNA reagent (Invitrogen) according to the manufacturer's instructions as a complementary method to evaluate the effect of the treatments on HT29 cells viability. The assay is based on the ability of the specific fluorochrome dye (PicoGreen) to make a stable complex with double-stranded DNA (dsDNA) instead of single-stranded DNA (ssDNA), proteins, SDS, and urea. This selective characteristic was used to monitor cell death, in which increases of dsDNA in the cell medium are proportional to increases in the fluorimetric signal intensity. The fluorescence was measured at an excitation of 480 nm and an emission of 520 nm recorded at room temperature (SpectraMax M2/M2e Multi-mode Plate Reader, Molecular Devices Corporation, Sunnyvale, CA, USA). These results were expressed as fluorescence values, which indicated the content of double-stranded DNA that was released into the environment as a result of cell death, as well as the percentage of DNA integrity compared with the negative control group treatment.

### 2.7. Genotoxicity Effect

The potential genotoxic effects of the treatments were evaluated using single cell gel electrophoresis analysis (DNA Comet assay), which was performed according to Singh and coworkers [18] and according to the general guidelines for the comet assay [19]. Freshly collected cells (10^6^) were treated as previously described for 24 h. The supernatant was removed and replaced with culture medium for 24 h. Next, 60 *μ*L of 0.6% low melting agarose (LMA) in PBS was added to the treated cells, and the cells were then transferred onto degreased microscope slides. One hundred cells (50 cells from each of two replicate slides) were selected and analyzed. The cells were visually scored according to the tail length and classified as follows: class 0 (absence of tail); class 1 (tail of up to 1× the diameter of the nucleus of negative control); class 2 (tail of up to 2× the diameter of the nucleus); class 3 (tail of up to 3× the diameter of the nucleus); and class 4 (tail of more than 3× the diameter of the nucleus). The scores ranged from 0 (no migration) to 4 (maximal migration). Thus, the damage index for cells ranged from 0 (all cells with no migration) to 400 (all cells with maximal migration). The slides were analyzed under blind conditions by at least two different individuals.

### 2.8. Assay for Caspases 8, 3, and 1 Activities

Caspases 1, 3 and 8 activities were determined using Fluorimetric Assay Kits (BioVision, Mountain View, CA). The fluorescence intensity of caspases 1, 3, and 8 was recorded at 400 nm and 505 nm for excitation and emission, respectively. The caspase activity was calculated as the fluorescence intensity (FI)/min/mL = ΔFlt/(*t* × *v*), where ΔFlt = difference in fluorescence intensity between time zero and time *t* minutes, *t* = reaction time in min, and *v* = volume of sample in mL. All results were obtained from six independent experiments. Protein content was determined according to Bradford [20].

### 2.9. Superoxide Dismutase (SOD) and Catalase (CAT) Activities

The cells were collected and adjusted to a concentration of 5 × 10^6^cells/mL in PBS buffer. CAT activity was determined according to the modified method described by Nelson and Kiesow [21]. The change in absorbance at 240 nm was measured for 2 min. CAT activity was calculated using the molar extinction coefficient (0.046 mM^−1^ cm^−1^), and the results were expressed as picomoles of CAT per milligram of protein.

The SOD activity was determined based on the inhibition of the radical superoxide reaction with adrenaline as previously described by McCord and Fridovich [22]. SOD activity was determined by measuring the rate of adrenochrome formation in medium containing glycine-NaOH (50 mM, pH 10) and adrenaline (1 mM), which was observed at 480 nm. The units of SOD are defined by the amount of enzyme that inhibits 50% of adrenaline oxidation and were expressed as units per 10^6^ cells.

### 2.10. Content ROS

The cells were collected and adjusted to a concentration of 1 × 10^6^cells/mL in PBS buffer. ROS production was estimated using the fluorescent probe, 2′,7′-dichlorofluorescein diacetate (DCFH-DA), as previously described by Ali and coworkers [23]. Briefly, the cells were homogenized and aliquots were incubated in the presence of DCFH-DA (5 *μ*m) at 37°C for 60 min. DCFH-DA was enzymatically hydrolyzed by intracellular esterases to form nonfluorescent DCFH, which was then rapidly oxidized to form highly fluorescent 2′,7′-dichlorofluorescein (DCF) in the presence of ROS. The DCF fluorescence intensity was proportional to the amount of ROS that was formed. Fluorescence was measured using excitation and emission wavelengths of 480 and 535 nm, respectively. A calibration curve was established with standard DCF (0.1 nm to 1 *μ*m), and the ROS levels were expressed as DCF mMol/mL.

### 2.11. RNA Extraction and qRT-PCR

Total RNA was isolated from cultured cells in the different treatments using Trizol (Invitrogen) following manufacturer's instructions. The RNA was quantified by absorbance at 260 nm. Total RNA (1 *μ*g) was treated with DNase (Invitrogen) at 37°C for 5 min to digest any contaminating DNA. The transcriptase reverse reaction was performed using iScript cDNA Synthesis Kit (Bio-Rad), according to manufacturer's instructions in a final volume of 20 *μ*L. Analysis of relative gene expression was performed by qRT-PCR using the StepOnePlus RT-PCR system (Applied Biosystems) with SYBR Select Master Mix (Applied Biosystems). Variability in the amount of mRNA was corrected by amplification of *β*-actin housekeeping gene.

The primers for ERCC1and for the housekeeping gene *β*-actin were designed in Primer Express software v 3.3 (Applied Biosystems) based on sequences available in GenBank. The oligonucleotides were synthesized by IDT (Integrated DNA Technologies). For the genes that have more than 1 transcript, all the sequences were aligned using ClustalW2 and the primer sequence was designed on the consensus region. The list of genes, forward primer, reverse primer, and GenBank accession number are as follows.

*ERCC1.* Consider CTCAAGGAGCTGGCTAAGATGTG; TTCTGCTCATAGGCCTTGTAGGT; NM_001166049.1; NM_001983.3; NM_202001.2. 

**β*-Actin*. Consider TGTGGATCAGCAAGCAGGAGTA; TGCGCAAGTTAGGTTTTGTCA; NM_001101.3.

### 2.12. Statistics

The data were evaluated using analysis of variance (ANOVA) and *t*-test and were expressed as a mean ± SD. *P* < 0.05 was considered statistically significant.

## 3. Results

### 3.1. Analysis of the Extract

**Figure 1:**
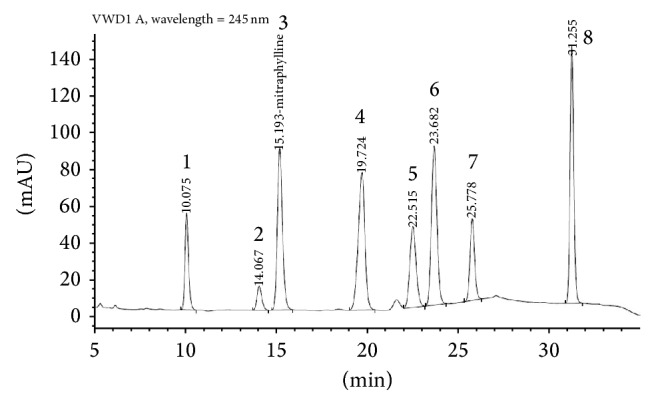
HPLC-fingerprint analysis of dry extract from* Uncaria tomentosa*. 1—Speciophylline (10.075 min), 2—uncarine F (14.067 min), 3—mitraphylline (15.193 min), 4—rhynchophylline (19.724 min), 5—isomitraphylline (22.515 min), 6—uncarine C (23.682 min), 7—isorhynchophylline (25.778 min), and 8—uncarine E (31.255 min).

The HPLC analysis of dry* Uncaria tomentosa* extract (Figure 1) has a content of 4.20% oxindole alkaloids (OA), quantified as the sum of speciophylline, uncarine F, mitraphylline, rhynchophylline, isomitraphylline, uncarine C, isorhynchophylline, and uncarine E.

### 3.2. Apoptosis Assay

**Figure 2:**
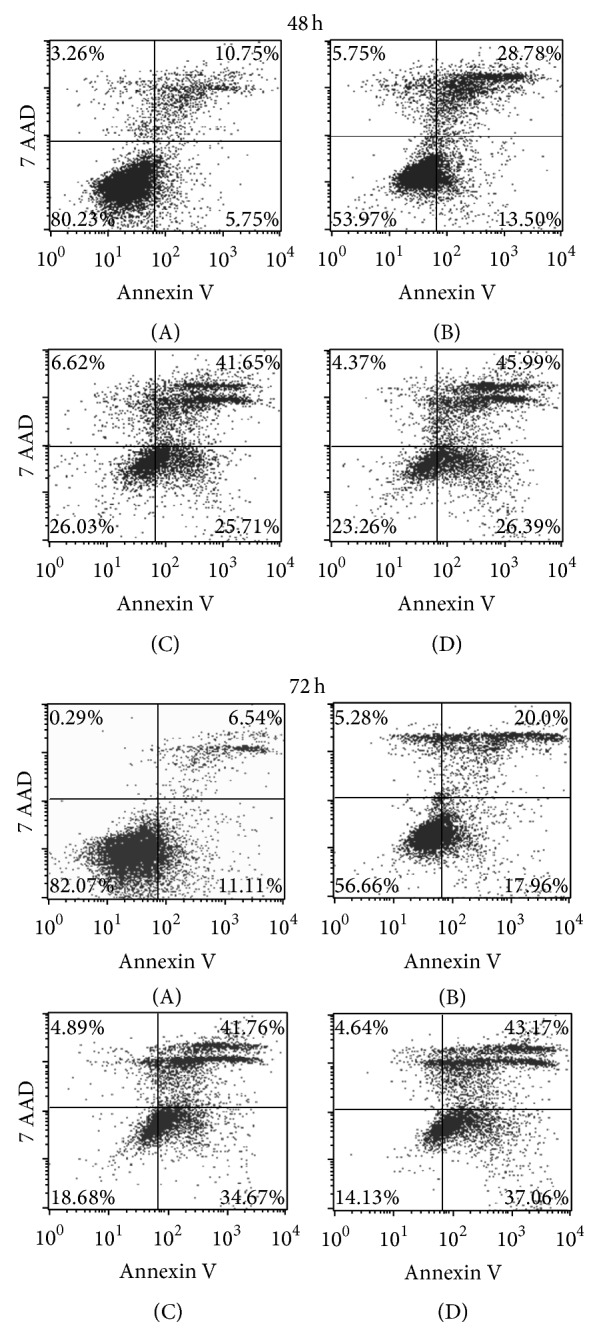
Apoptosis was assessed using PE Annexin V Apoptosis Detection Kit I (BD Pharmingen). The cells were treated with the following concentrations of cytotoxic agent for 48 h or 72 h: (A) = control: culture medium without* Uncaria tomentosa *extract or oxaliplatin; (B) = chemotherapy: oxaliplatin 20 *μ*mol/L; (C) =* Uncaria*:* Uncaria tomentosa* extract 750 *μ*g/mL; (D) =* Uncaria* + chem.:* Uncaria tomentos*a extract 750 *μ*g/mL + oxaliplatin 20 *μ*mol/L. A total of 10,000 events were acquired and analyzed using FACSCalibur with CELLQuest software. Cells PE Annexin V and 7-AAD negative were considered viable cells; live-gated cells within the Annexin-V^+^7AAD^−^ compartment were identified as early apoptotic cells and gated cells within the Annexin-V^+^7AAD^+^ compartment were identified as late apoptotic/dead cells.

A marked reduction in the number of viable cells (Annexin-V^−^/7AAD^−^) after either* Uncaria tomentosa* or oxaliplatin was observed. Importantly,* Uncaria tomentosa* induced increased apoptosis in HT29 tumor cells at 48 h of exposure compared with control (Annexin V^+^ cells: 67% × 16%). Similar results were observed in combined* Uncaria* + chemotherapy treatment.* Uncaria tomentosa* and* Uncaria* + chemotherapy exposure for 72 h resulted in major apoptotic cell death, with a higher percentage of 7AAD^+^ cells, indicating not only an increase of early apoptotic (Annexin-V^+^/7AAD^−^) but also an increase in late apoptotic/necrotic cells (Annexin-V^+^/7AAD^+^) (Figure 2).

### 3.3. Comet Assay

**Figure 3:**
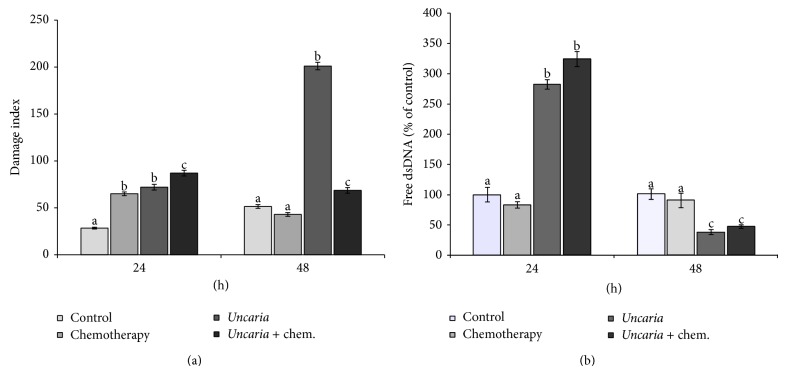
Single cell gel electrophoresis analysis (DNA Comet assay) (a) and PicoGreen assay (b). HT 29 cells were seeded in 96-well flat bottom plates at 1 × 10^6^ viable cells/mL and were allowed to adhere at 37°C overnight. The cells were treated with the following concentrations of cytotoxic agent for 24 h: control = culture medium without* Uncaria tomentosa *extract or oxaliplatin; chemotherapy = oxaliplatin 20 *μ*mol/L;* Uncaria* =* Uncaria tomentosa* extract 750 *μ*g/mL;* Uncaria* + chem. =* Uncaria tomentos*a extract 750 *μ*g/mL + oxaliplatin 20 *μ*mol/L. The supernatant was removed and replaced with culture medium for over 24 h (named 48 h). Different lowercase letters represent statistically significant differences among the treatments (*P* < 0.05).

We determined the DNA integrity in single cells using the comet assay. Chemotherapy treatment induced apoptosis in a few cells, which showed an increase in the tail after 24 h of treatment (classes 1, 2, 3, and 4, data not shown) in comparison with the control.* Uncaria tomentosa *treatment also induced apoptosis (class 4), but with fewer cells in classes 2 and 3, resulting in a damage index similar to that observed after chemotherapy treatment and less than that after the combination of the two cytotoxic agents. After removal of the supernatant and further incubation for 24 h (referred to as 48 hs), the events observed in treatments with* Uncaria tomentosa* and* Uncaria* + chemotherapy were not reversed, indicating that an even higher percentage of cells were induced to undergo apoptosis. This was not the case in cells exposed to oxaliplatin, in which the cytotoxic effects were partially reversed, as observed by changes in the damage index (Figure 3).

### 3.4. PicoGreen Assay

PicoGreen dye is a fluorescent nucleic acid stain for quantification of double-stranded DNA (dsDNA). This assay is based on the principle that a greater percentage of free dsDNA in the supernatant results from an increased number of apoptotic cells, thereby indicating cytotoxicity. We found that, within 24 h,* Uncaria tomentosa* induced major cytotoxicity when used alone or in combination with chemotherapy as compared with chemotherapy alone. However, at 48 h we did not observe an increase in cell death, which was potentially due to the relative lack of intact cells once most of the cells were injured at 24 h (Figure 3).

### 3.5. Caspase Activities

**Figure 4:**
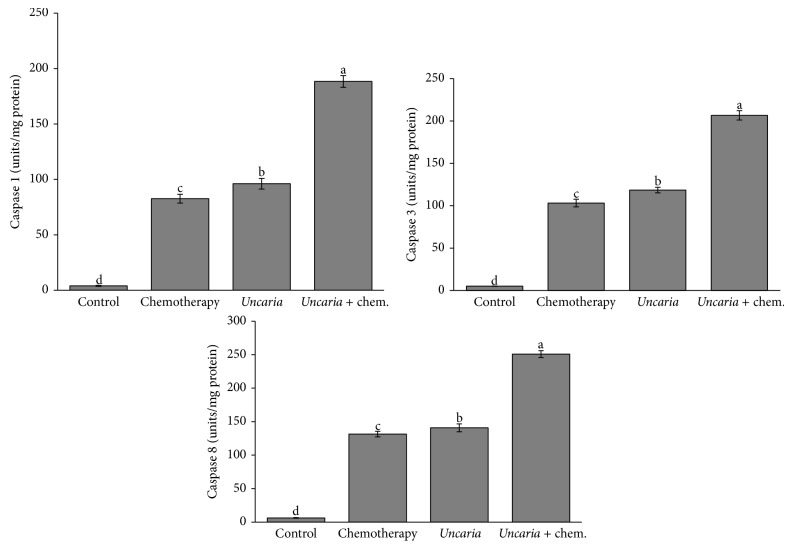
Caspase activities. It was determined using Fluorimetric Assay Kits. HT 29 cells were seeded in 96-well flat bottom plates at 1 × 10^6^ viable cells/mL. The cells were treated with the following concentrations of cytotoxic agent for 48 h: control = culture medium without* Uncaria tomentosa* extract or oxaliplatin; chemotherapy = oxaliplatin 20 *μ*mol/L;* Uncaria* =* Uncaria tomentosa* extract 750 *μ*g/mL;* Uncaria* + chem. =* Uncaria tomentosa* extract 750 *μ*g/mL + oxaliplatin 20 *μ*mol/L. Different lowercase letters represent statistically significant differences among the treatments (*P* < 0.05).

*Uncaria tomentosa* extract and oxaliplatin significantly induced increased activity of caspases 8 and 1 and of the effector caspase 3 when compared with control.* Uncaria tomentosa* extract induced a higher caspase activity than oxaliplatin, and synergy was observed when* Uncaria tomentosa* was added to oxaliplatin for induction of all caspase activities (Figure 4).

### 3.6. SOD, CAT Activity, and ROS Content

**Figure 5:**
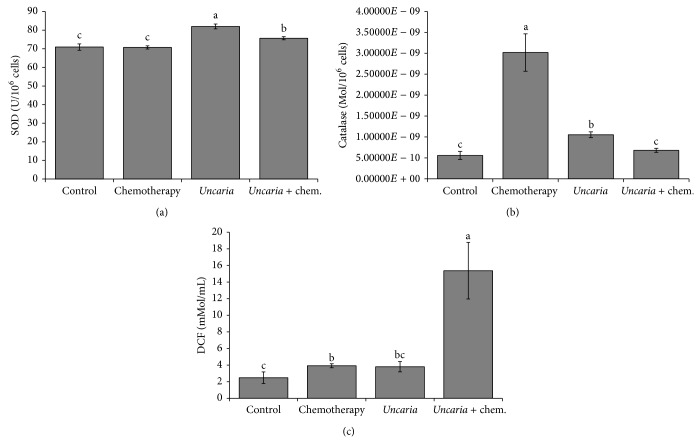
Oxidative stress. HT 29 cells were seeded in 6-well flat bottom plates at 1 × 10^6^ viable cells/mL. The cells were treated with the following concentrations of cytotoxic agent for 24 h (DCF test) or 48 h (SOD and catalase activity): control = culture medium without* Uncaria tomentosa* extract or oxaliplatin; chemotherapy = oxaliplatin 20 *μ*mol/L;* Uncaria* =* Uncaria tomentosa* extract 750 *μ*g/mL;* Uncaria* + chem. =* Uncaria tomentosa* extract 750 *μ*g/mL + oxaliplatin 20 *μ*mol/L. The cells were collected and adjusted to a concentration of 1 × 10^6^ cells/mL in PBS buffer. (a) SOD activity: the units of SOD are defined by the amount of enzyme that inhibits 50% of adrenaline oxidation and were expressed as units per 10^6^ cells. (b) Catalase activity was calculated using the molar extinction coefficient (0.046 mM^−1^ cm^−1^), and the results were expressed as picomoles of catalase per 10^6^ cells. (c) ROS production was estimated using the probe DCFH-DA, which is oxidized to fluorescent DCF. Different lowercase letters represent statistically significant differences among the treatments (*P* < 0.05).

Treatment with* Uncaria tomentosa* extract at a concentration of 750 *μ*g/mL significantly increased the activity of SOD, while the addition of oxaliplatin resulted in an increase of the enzyme catalase (Figure 5). The ROS content was significantly increased in comparison with the control with treatment of the* Uncaria tomentosa* extract plus oxaliplatin and did not have differences between* Uncaria tomentosa* and oxaliplatin treatments (Figure 5).

### 3.7. Expression of ERCC1 mRNA

**Figure 6:**
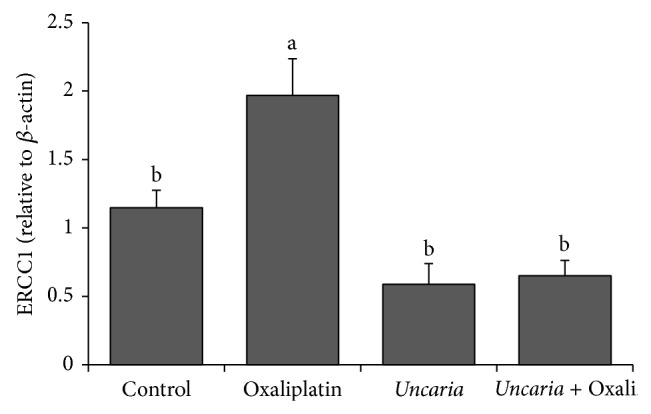
ERCC1 mRNA level-HT29 cell was treated with oxaliplatin (20 *μ*M),* Uncaria tomentosa* extract (250 *μ*g/mL), and* Uncaria tomentosa* + oxaliplatin for 48 h. Total RNA was isolated and subjected to qRT-PCR. Different lowercase letters represent statistically significant differences among the treatments (*P* < 0.05).

The treatment with oxaliplatin resulted in increase of expression of ERCC1 mRNA.* Uncaria tomentosa* extract resulted in downregulation of expression of ERCC1, induced by oxaliplatin exposure, once exposure of* Uncaria tomentosa* combined with oxaliplatin significantly decreased ERCC1 mRNA levels in relation to the treatment with oxaliplatin alone (Figure 6).

## 4. Discussion

### 4.1. OA Content of the* Uncaria tomentosa *Extract and Apoptosis

Two subt ypes of oxindole alkaloids, pentacyclic oxindole alkaloids (POA) and tetracyclic oxindole alkaloids (TOA), have been identified in* Uncaria tomentosa*, which indicates the existence of two chemotypes of* Uncaria tomentosa*. One chemotype contains POA and the other chemotype contains TOA in addition to POA and tetracyclic indole alkaloids in various parts of the plant [7]. In 1998, Wurm and coworkers [24] showed that the supernatant from endothelial cells cultured with* Uncaria tomentosa* POA (mitraphylline/isomitraphylline and isopterodine) had an antiproliferative effect on T and B lymphoblastoid cells (Jurkat and Raji cells). This activity was inhibited in a dose-dependent manner by the addition of TOA (rhynchophylline and isorhynchophylline). The extract in the present study is composed of TOA and POA, which did not affect its ability to induce apoptosis on neoplastic cells when used directly in the culture medium at a concentration higher than that reported by Wurm and coworkers [24]. The apoptotic effect of* Uncaria tomentosa* on neoplastic cells has been reported in several studies [12, 15]. Pilarski and coworkers [12] used an extract derived from 10% of the bark in water or ethanol (50% or 96%) and reported an IC_50_ ranging from 499 to 803 *μ*g/mL in HT29 cells. We used a 5% extract in 70% ethanol at a concentration of 750 *μ*g/mL and also observed a significant apoptotic effect. These results were observed in an Annexin V and 7AAD assay using flow cytometry, as well as by caspase activity and the comet assay. The main objective of this study was to evaluate the interaction between oxaliplatin and* Uncaria tomentosa*. Supplementation with herbal products with antioxidant effects in cancer patients remains controversial. The beneficial effects derive from use of antioxidants to mitigate toxicity and thus enable uninterrupted treatment schedules and a reduced need for lower chemotherapy doses. However, if reactive oxygen species are necessary for the activity of specific chemotherapy drugs, then use of antioxidants could be undesirable [25]. We did not observe interference of the* Uncaria tomentosa* extract in the cytotoxic activity of oxaliplatin, and our data demonstrated an apoptotic effect of* Uncaria tomentosa* in adenocarcinoma cells. However, it is necessary to consider the potential metabolic interaction of these drugs.* Uncaria tomentosa* inhibits* in vitro* metabolism of drug marker substrates via human cytochrome P-450 (CYP).* Uncaria tomentosa* is moderately inhibitory (>56%) to isoform 3A4 and slightly inhibitory against other isoforms (2C9, 2C19, and 2D6) [26]. The substrates that are metabolized by the CYP3A isoform include the chemotherapy drugs dasatinib, cyclosporine [27], irinotecan, and oxaliplatin [28]. Inhibition of the metabolic enzymes could result in enhanced toxicity. This should be considered if* Uncaria tomentosa* is clinically used in combination with these chemotherapy agents [10, 29] to minimize side effects such as neutropenia.

### 4.2. Caspase Activities

Anticancer drugs kill tumor cells via activation of apoptotic pathways through two major signaling pathways: the mitochondrial or intrinsic pathway and the death-receptor or extrinsic pathway [30]. Extrinsic pathway factors, including death receptors and a death domain-containing adaptor protein, stimulate apoptosis via the activation and binding of ligands and prodeath caspases [31]. Eight members of the death receptor family have been characterized, including tumor necrosis factor receptor 1 (TNFR1; also known as DR1), CD95 (also known as DR2, APO-1, and Fas), and DR3 (also known as APO-3). To study the apoptotic effect of* Uncaria tomentosa *and oxaliplatin, we analyzed the extrinsic pathway of apoptosis by evaluating the activities of caspase 8 (FLICE), caspase 1 (interleukin-1*β* converting enzyme, ICE), and caspase 3 (CPP32). Treatment with* Uncaria tomentosa *not only caused a greater activity of the three caspases examined but also showed a further increase in activity when added with oxaliplatin in the medium. The TNF receptor and ligand family and death effector domain family are activated by oxaliplatin treatment, and caspases are the principal genes leading to treatment response to oxaliplatin [32]. Caspase 8, an initiator caspase, is activated by the CD95 and TNFR1 pathway and subsequently activates the effector caspase 3 [33], inducing irreversible apoptosis in the cells. Although caspase 1 is associated with inflammatory processes, it participates in apoptosis via the extrinsic pathway through death receptor DR3, which activates apoptosis via the initiator caspase 8. This subsequently induces downstream molecular events by activation of either the executioner caspase 3 or caspase 1 [34].

Bacher and coworkers [15] previously demonstrated that apoptosis due to* Uncaria tomentosa* POA (pterodine) did not occur via the CD95/Fas pathway in T lymphoblastic cells. It is interesting that apoptosis also occurs via mechanisms other than the CD95 pathway because many carcinomas express CD95 at abnormally low levels or lack expression completely. Moreover, colon carcinoma cell lines are relatively resistant to CD95-mediated apoptosis [35]. Caspase 1 has also been implicated in resistance to Fas-mediated apoptosis in colon cancer cells in which it is downregulated, and sensitization of these cells to Fas via IFN-*γ* treatment coincides with an upregulation of caspase 1 [36].

Gonçalves and coworkers [37] have also reported that antimicrotubule agent-induced apoptosis involves caspase 8 activation. This apoptotic pathway is independent of CD95 ligation in HT29-D4 cells. They demonstrated that caspase 8 activation might be a common signaling event for several apoptotic pathways independent of the contribution of CD95. Subsequently, Lacour and coworkers [38] showed that exposure of colon carcinoma cells to various cytotoxic drugs induced the formation of a CD95-including death-inducing signaling complex (DISC) in a ligand-independent manner. This caused a redistribution of CD95 receptor into lipid rafts at the surface of HT29 cells, thus allowing cytotoxic drugs to synergize with death receptor ligands and induce tumor cell death. They also demonstrated that FADD and procaspase 8 are recruited into rafts following cisplatin treatment.

Our data are consistent with other studies. The induction of apoptosis in cancer cells via caspases 3 and 8 by* Uncaria tomentosa* extract has been previously described by de Martino et al. [14] and Cheng and coworkers [13]. However, more studies are required to assess whether the activation of caspase 8 by* Uncaria tomentosa* extract or oxaliplatin also occurs through the death receptor DR3 in addition to the already described activation FAS/APO1 pathway [13].

### 4.3. Oxidative Stress

Laurent and coworkers [39] demonstrated that ROS can exert differential effects between normal cells and tumor cells with respect to proliferation versus apoptosis and thus that antioxidants can play different roles in a cell type-dependent manner. In certain contexts, products that reduce levels of H_2_O_2_ inhibit the proliferation of normal cells and increase tumor cell proliferation. However, antioxidants that mimic the effect of the enzyme SOD increase the levels of H_2_O_2_ via superoxide anion dismutation and induce apoptosis of tumor cells* in vitro* and exert an* in vivo* antitumor effect. However, in this study they observed a synergistic effect of oxaliplatin with SOD mimics [39].

Alexandre and coworkers [40] subsequently reported that two mimics of SOD (CuDIPS; MnTBAP) and Mangafodipir (chelates manganese with SOD-, catalase-, and glutathione reductase-like properties) increased the cytotoxicity of oxaliplatin, paclitaxel, and 5-fluorouracil in CT26 colon cancer cells in a dose-dependent manner through increased H_2_O_2_ accumulation. Conversely, N-acetylcysteine decreased the cytotoxicity exerted by these chemotherapies [40]. Thus, the increase in SOD activity observed in this study after treatment with* Uncaria tomentosa* extract may contribute to the cytotoxicity of the chemotherapeutic. Our data are consistent with those of Pilarski and coworkers [9], which showed that ethanol extract of* Uncaria tomentosa* had increased superoxide radical scavenging activity (SOD-like activity). The addition of* Uncaria tomentosa* extract plus oxaliplatin resulted in increased levels of ROS compared with the control, which might be correlated to the increased activity of SOD. The basal concentration of reactive oxygen species (particularly H_2_O_2_) is higher in cancer cells compared with normal counterparts, due to a higher production of superoxide anions via the respiratory chain and cytoplasmic NADPH oxidase [41]. Thus, an increase in Mn-SOD activity could potentially cause H_2_O_2_-induced cytotoxicity and contribute to apoptosis, as observed in a study using natural polyphenols [41]. However, the modest increase in the activity of SOD and CAT enzymes alone is not sufficient to explain the major increase in the oxidizate DCF that was observed. Karlsson and coworkers [42] showed that significant cytosolic oxidation of H2DCF to DCF depends on the combined effect of Fenton-type reactions (mediated by ROS) and enzymatic activity of cytochrome c. It further depends on the release of redox-active iron to the cytosol and/or mitochondrial release of cytochrome c for the induction of a strong cytosolic DCF-mediated fluorescence, such as that which was observed in present study. So, the strong fluorescence observed in cells treated with* Uncaria tomentosa* plus oxaliplatin may also reflect the large percentage of cells undergoing apoptosis and consequently releasing cytochrome c (due to activation of caspases).

### 4.4. DNA Repair

Oxaliplatin covalently binds to DNA and interferes with DNA replication by inducing production of DNA adducts and causes both intra- and interstrand cross-links. In mammalian cells, DNA intrastrand cross-links are repaired by the nucleotide excision repair (NER) system. The removal of these adducts from genomic DNA is mediated by an enzyme called excision repair cross-complementing 1 (ERCC1), a structure-specific DNA repair endonuclease that is responsible for incision at the 5′ site of damaged DNA. In a previous study, we demonstrated that the extract of* Uncaria tomentosa* restored the damage caused by oxaliplatin using cytogenetic analysis in an* Allium cepa* test. This did not occur in present study with HT29 cells. This result is beneficial, as increased DNA repair activity is associated with a poorer response to treatment. ERCC1 may affect the survival time after treatment with oxaliplatin [43]. Arnould and coworkers [44] showed that while the residual level of Pt-DNA adducts was related to cisplatin cytotoxicity, this did not occur for oxaliplatin. Repair of the lesions induced by oxaliplatin and the manner by which cells address the mutations induced by translesion synthesis are more important factors for cytotoxicity than the level of DNA adducts [44].

Resistant human colorectal cancer cell lines (among them the HT 29) show a significant induction in mRNA and protein levels of ERCC1 when exposed to oxaliplatin, which is in contrast to what occurs with sensitive cell lines [45]. Mallick and coworkers [46] showed that the induction of ERCC1 is more important than baseline ERCC1 expression as a marker of oxaliplatin resistance in human colorectal cancer cells lines. Seetharam and coworkers [45] further showed that this gene expression is reversible by targeted suppression of ERCC1.

The results of our study show that* Uncaria tomentosa* extract induces apoptosis in HT29 cells without overexpression of the mRNA ERCC1, as opposed to what is observed with oxaliplatin treatment.* Uncaria tomentosa* extract resulted in downregulation of expression of mRNA ERCC1 in HT29 cells exposed to oxaliplatin. Certain natural products such as curcumin (natural phenolic compound) and emodin (natural anthraquinone) enhance the sensitivity of chemotherapeutic treatment for non-small-cell lung cancer (NSCLC) by decreasing ERCC1 protein levels [47, 48]. In these studies, the combination of cisplatin and curcumin or emodin produced significantly lower ERCC1 mRNA levels when compared with cisplatin alone, indicating that these products can downregulate both protein and mRNA expression of ERCC1 in these human lung cancer cell lines. Depletion of endogenous ERCC1 expression significantly enhanced lung cancer cell death after treatment with cisplatin [47, 48].

## 5. Conclusion

*Uncaria tomentosa* extract did not reduce the cytotoxic effects of chemotherapy.* In vitro*, the concomitant use of* Uncaria tomentosa* and oxaliplatin resulted in increased levels of apoptosis in adenocarcinoma cells via an increase in the activities of caspases 8, 3, and 1 and decreased ERCC1 mRNA levels. Taken together,* Uncaria tomentosa* extract may be useful for cancer patients as a complementary and alternative medicine, making sure not to interfere with the well-established treatment protocols, as well as evaluating the possible CYP3A4 inhibition. An alternative would be the sequential use of the herbal product after the chemotherapy procedure. Further studies are required to evaluate whether this beneficial effect extends to other chemotherapeutic drugs.
